# Optimizing treatment of lepromatous form of leprosy using ofloxacin on top of standard multi-drug therapy in National Referral Hospital, Jakarta, Indonesia

**DOI:** 10.12688/f1000research.161758.1

**Published:** 2025-03-03

**Authors:** Mufqi Handaru Priyanto, Malika Sabrina Yunifananda, Sri Linuwih SW Menaldi, Erni Juwita Nelwan, Melani Marissa

**Affiliations:** 1 Department of Dermatology and Venereology, Faculty of Medicine, Universitas Indonesia, Depok, West Java, Indonesia; 2 Universitas Indonesia Hospital, Depok, West Java, Indonesia; 3 Rumah Sakit Dr Cipto Mangunkusumo, Central Jakarta, Jakarta, Indonesia; 4 Department of Internal Medicine, Faculty of Medicine, Universitas Indonesia, Depok, West Java, Indonesia

**Keywords:** Lepromatous Leprosy; Ofloxacin; Multi-Drug Therapy (MDT); Bacteriological Index (BI); Morphological Index (MI); Leprosy Treatment Optimization; Indonesia

## Abstract

**Background:**

Standard multi-drug therapy (MDT) with duration of 6-12 months is generally effective for treating leprosy. However, in cases of lepromatous (LL) and borderline lepromatous (BL) patients with high bacterial loads and complicated circumtances, prolonged duration treatment is more often observed. As prolonged duration may affect patient adherence negatively, adding ofloxacin to MDT is a promising approach to prevent prolonged treatment.

**Method:**

This retrospective cohort study investigated the effects of adding ofloxacin to MDT in 21 patients diagnosed with LL or BL leprosy at Cipto Mangunkusumo National Referral Hospital, Jakarta, Indonesia. Bacterial load and viability were tracked using the Bacteriological Index (BI) and Morphological Index (MI), and were compared using non-parametric Friedman test before and after the patients were given ofloxacin.

**Findings:**

Adding ofloxacin to MDT led to a significant reductions in both BI and MI. The median MI dropped to zero after six months of combined treatment (p<0.001), with significant differences between baseline and 6, 9, and 12-months. BI also significantly declined (p=0.007), with significant reductions between baseline and 3, 6, 9, and 12-month assessments. The proportion of patients reaching an MI of zero also steadily increased.

**Interpretation:**

Ofloxacin as an adjunctive therapy to MDT substantially improves treatment of leprosy with high bacterial and morphological index. Faster bacterial clearance prevent prolonged treatment duration, potentially improving adherence, outcomes and reducing relapse risk. Ofloxacin is the only second-line leprosy treatment covered by the national health insurance in Indonesia. Earlier initiation of this adjunctive therapy may offer greater benefits.

## Introduction

Despite global efforts to eliminate Leprosy, Indonesia continues to be a country with a high prevalence of the disease, with 17,251 cases reported by the Indonesian Ministry of Health in 2023. The World Health Organization (WHO) recognizes Indonesia as having one of the highest leprosy burden worldwide. ^1^ In 2024, a total of 413 leprosy cases have been registered at Dr. Cipto Mangunkusumo National Hospital. ^2^ The more severe and contagious form of leprosy – lepromatous leprosy (LL) and borderline lepromatous leprosy (BL) – are especially concerning due to their high bacterial loads and may lead to significant morbidity if not properly treated. ^3^ Since 1981, multi-drug therapy (MDT) combining rifampicin, dapsone, and clofazimine by the World Health Organization (WHO) has been used as the standard treatment for leprosy. ^4^

While MDT is effective, its use alone can lead to prolonged treatment durations, particularly in patients with high bacterial loads, which may contribute to poor adherence. Adding medication to the current regimen could be considered to improve patient’s adherence. ^5^ Recently, more studies have analyzed the potential of additional antibacterial agents in enhancing the efficacy of MDT for leprosy cases. Ofloxacin, a fluoroquinolone with strong activity against *M. leprae*, has shown promise when added to standard MDT. One study highlighted the potential benefits of using ofloxacin in leprosy treatment, noting that it is well tolerated and achieves similar bacterial clearance, even when ofloxacin is only administered during the first month. ^6^ A retrospective study further demonstrated that patients receiving MDT combined with ofloxacin showed good adherence, as no adherence failures was identified among subjects receiving ofloxacin. Additionally, the combination therapy was associated with a lower relapse rate and improved long-term outcomes, supporting its use as a promising option for treating severe leprosy cases. ^5^

The evaluation of leprosy treatment is typically followed up using two key bacteriological indices: the Bacterial Index (BI), which measures the overall bacterial load in skin smears, and the Morphological Index (MI), which assesses the proportion of viable (solid) bacilli, which depicts treatment efficacy. A significant reduction in both indices over time reflects successful bacterial clearance and a reduced risk of disease transmission and relapse. ^7^

Certainly, there are specific considerations that should be given while adding ofloxacin to standard MDT treatment, such as: initial high viable infection load that is indicated by a high MI from acid-fast staining of slit-skin smears, persistently positive MI after at least six months therapy of standard MDT, ^8^ or relapse, diagnosed based on criteria by Linder et al. ^9^

With above mentioned findings, this study aims to analyse the efficacy of MDT combined with ofloxacin in patients with lepromatous form of leprosy (BL and LL). By assessing the reduction in BI and MI during the course of treatment regimens, the study seeks to determine whether adding ofloxacin enhances MDT’s bacteriological outcomes, leading to faster bacterial clearance and improved patient outcomes. This is particularly important for patients with lepromatous form of leprosy, where the bacterial load is high and conventional MDT may take longer time than standard duration of multibacillary (MB) therapy to achieve bacteriological cure.

## Methods

### Study design

Between 2020 and 2024, all medical records of eligible patients with lepromatous form leprosy treated at the Dermatology and Venereology Clinic, Dr. Cipto Mangunkusumo National Hospital, Jakarta, Indonesia, were examined in this retrospective cohort study.

The cohort study evaluated treatment outcomes by comparing BI & MI parameters in the same patients before and after the initiation of ofloxacin 400 mg once daily as an adjunct therapy.

### Participants

**Figure 1.**
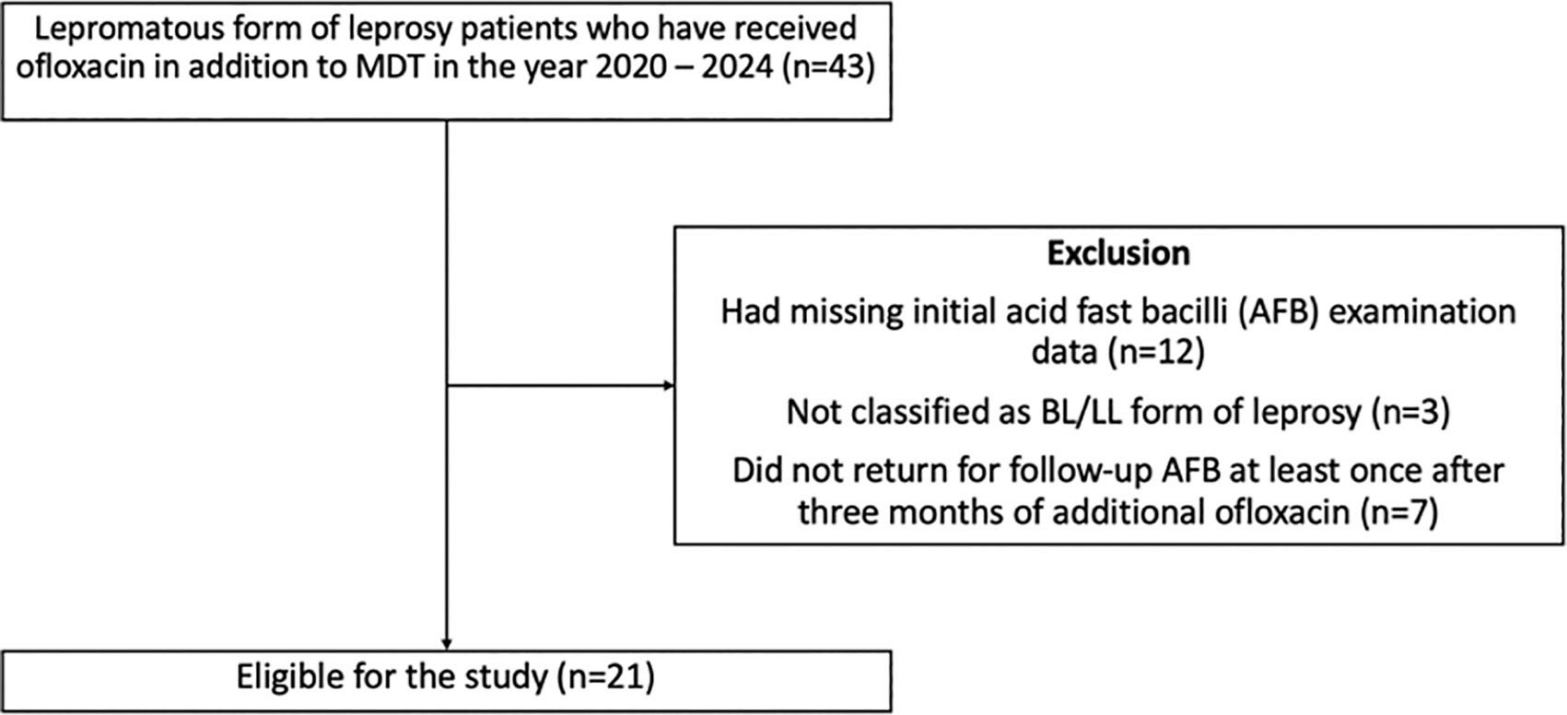
Selection of participants for the study.

Patients were included if they (1) were diagnosed with leprosy according to WHO criteria; (2) classified as borderline lepromatous (BL) leprosy or lepromatous form of leprosy (LL) based on Ridley-Jopling criteria ^10^; (3) were adults aged ≥18 years at the start of leprosy treatment; and (4) received ofloxacin in addition to the standard multidrug therapy (MDT) regimen under these circumstances: (a) a high initial viable infection load, evidenced by a high MI from acid-fast staining of slit-skin smears; (b) persistently positive MI, typically after ≥6 months of standard MDT; (c) re-positivity of MI; and (d) relapse, diagnosed based on criteria by Linder et al. ^8, 9^ To prevent selection biases, patients were excluded if they (1) had missing initial AFB examination data or (2) did not return for follow-up acid-fast bacilli (AFB) examination at least once after three months of additional ofloxacin ( Figure 1).

### Procedures

Patient data were extracted from electronic patients records by authors on October 2024. Patient information related to demographic, clinical presentation, disability status based on WHO disability grading (grade 0 for no disability, grade 1 for loss of sensation but no visible deformity or damage, and grade 2 for visible deformity of damage present), treatment course, microbiology investigations particularly the results from slit-skin smear examination, and also treatment outcome were gathered.

At our center, slit-skin smear examinations are routinely performed from six sites (both earlobes and four lesions) every three months in patients with positive initial MI or in cases requiring close monitoring. Smears are processed using Ziehl-Neelsen staining and evaluated for bacterial and morphological indices by a trained technician, under the supervision of a clinical microbiologist. The BI represents the total number of bacilli, measured semi-quantitatively using the Ridley-Jopling logarithmic scale, while the morphological index is the percentage of solid-staining bacilli.

After commencing ofloxacin therapy, patients underwent follow up to do acid-fast bacilli (AFB) examination at least once after three months of addition of adjunct therapy. At each visit, dermatology resident performed clinical examination, including assessment for new lesions, nerve involvement and other clinical findings. Slit-skin smears were collected from standard sites to determine the BI and MI according to established procedures.

### Outcomes

The primary outcomes of this study are (1) change in Bacteriological Index (BI) as this measures the bacterial load in skin smears, and (2) change in Morphological Index (MI) for assesses the proportion of viable bacteria in the smears. The secondary outcome of this study is a proportion of patients achieving an MI of zero.

### Statistical analysis

Statistical analyses were conducted using SPSS Statistics 26 (IBM Corp, Armonk, NY, USA). and visualized using GraphPad Prism 9.0 (GraphPad Software, Inc., San Diego, CA, USA). Spatial data was visualized using Google My Maps (Google LLC, Mountain View, CA, USA).

Subject characteristics were presented as frequency and percentage for categorical data, while numerical data were reported as mean and standard deviation. The non-parametric Friedman test was performed to compare MI and BI across AFB measurements, with post-hoc Dunn’s test applied to identify significant differences between time points and baseline value.BI across AFB measurements, with post-hoc Dunn’s test applied to identify significant differences between time points and baseline value.

## Results

### Sociodemographic and disease characteristics

**Figure 2.**
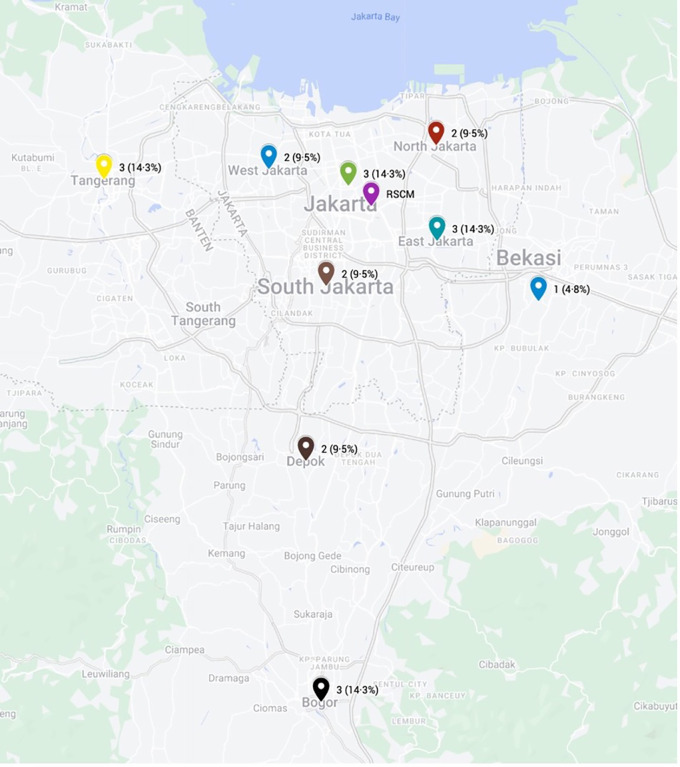
Patients’ domicile.

Twenty-one patients met the eligibility criteria and were included in the analysis ( Table 1). The majority of the subjects were male (66.7%) and unemployed (42.9%). All patients resided in the Jakarta metropolitan area, with most domiciled in Jakarta province (57.2%) and the remainder in surrounding regions (Tangerang, Bogor, Depok, and Bekasi) ( Figure 2). According to the Ridley-Jopling classification, most patients (71.4%) had polar lepromatous (LL) leprosy, while the remaining patients had borderline lepromatous (BL) disease. Notably, one LL patient was diagnosed with histoid leprosy, and another had Lucio’s leprosy. In terms of disability, 11 patients (52.4%) had no disability, 3 patients (14.3%) had grade 1 disability, and 7 patients (33.3%) had grade 2 disability. Additionally, 6 patients (28.6%) had no history of reaction, while 14 patients (66.7%) experienced a type 2 reaction, 1 patient (4.8%) had a type 1 reaction, and 1 patient (4.8%) had Lucio’s phenomenon. Patients received WHO-MDT for a duration of 9 months (median; range 1–31 months) before starting combined therapy with ofloxacin.

**Table 1.** Sociodemographic and disease characteristics of the subjects.

Characteristics	Values
Age	35.3 ± 11.9
Sex	
Male	14 (66.7%)
Female	7 (33.3%)
Occupation	
Unemployed	9 (42.9%)
Private-sector employee	5 (23.8%)
Student	3 (14.3%)
Self-employed	2 (9.5%)
Others	2 (9.5%)
Domicile	
Jakarta Special Capital Region	12 (57.2%)
Central Jakarta	3 (14.3%)
East Jakarta	3 (14.3%)
South Jakarta	2 (9.5%)
West Jakarta	2 (9.5%)
North Jakarta	2 (9.5%)
Tangerang	3 (14.3%)
Bogor	3 (14.3%)
Depok	2 (9.5%)
Bekasi	1 (4.8%)
Ridley-Jopling classification	
Borderline lepromatous (BL)	6 (28.6%)
Polar lepromatous (LL)	15 (71.4%)
Disability	
Grade 0	11 (52.4%)
Grade 1	3 (14.3%)
Grade 2	7 (33.3%)
Reaction/history of reaction	
None	6 (28.6)
Type 1	1 (4.8)
Type 2	13 (61.9)
Lucio’s phenomenon	1 (4.8)

### Morphological index (MI) clearance on MDT combined with ofloxacin

**Figure 3.**
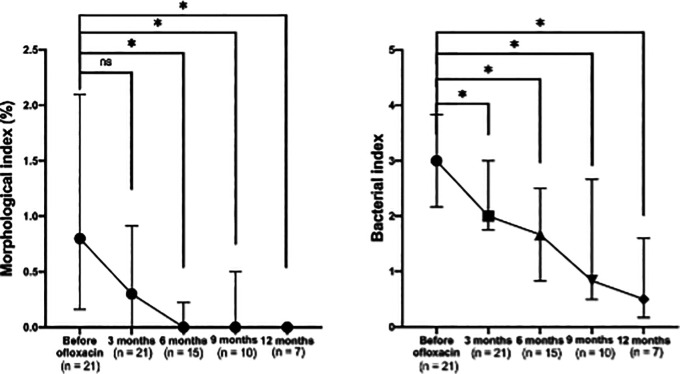
Changes in absolute morphological (left) and bacterial (right) indices before and after ofloxacin addition.

A significant reduction in the morphological index (MI) was observed after the addition of ofloxacin to the standard MDT regimen ( Figure 3, left). The median MI decreased to 0 after six months of ofloxacin treatment. Significant differences were noted across visits (p < 0.001), with post-hoc comparisons showing significant differences between baseline and six months (p = 0.003), nine months (p = 0.014), and 12 months (p = 0.008), but not at three months (p = 0.060).

### Bacterial index (BI) clearance on MDT combined with ofloxacin

A consistent and significant reduction in the bacterial index (BI) was also observed following the addition of ofloxacin ( Figure 3, right). Significant differences were noted across visits (p = 0.007), with pairwise comparisons showing significant reductions in BI between baseline and three months (p = 0.039), six months (p = 0.003), nine months (p = 0.006), and 12 months (p = 0.004).

### Proportion of patients achieving MI 0

**Figure 4.**
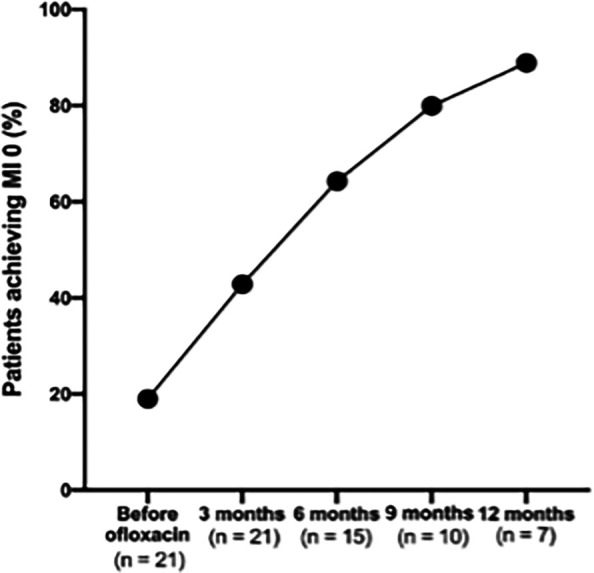
Proportion of patients achieving absolute morphological index of 0 before and after the addition of ofloxacin.

There was a consistent increase in the proportion of patients achieving an MI to 0 following the addition of ofloxacin, demonstrating the treatment’s effectiveness in reducing viable bacterial load over time ( Figure 4).

## Discussion

This study demonstrated a significant reduction in the morphological index (MI) following the addition of ofloxacin to the standard MDT regimen, and the median MI also decreased to 0 after six months of ofloxacin treatment. This finding aligns with the known bactericidal effects of ofloxacin, a floroquinolone antibiotic that directly kills the bacteria responsible for leprosy rather than merely inhibiting its growth. ^11^ This characteristic makes ofloxacin highly effective in reducing the bacterial load in leprosy patients, leading to faster clinical improvements and reducing transmission risk. Moreover, ofloxacin has proven effective in both paucibacillary (PB) and multibacillary (MB) forms of leprosy, especially while combined with other potent bactericidal agents like rifampicin and minocycline. ^12^ While ofloxacin-based multidrug therapy (OMDT) has a similar treatment duration, the fluoroquinolone component may provide better efficacy in the lepromatous form of leprosy cases, as mentioned in the inclusion criteria in this study about clinical indications for initiating ofloxacin, that might require more aggressive approach to achieve high cure rate. ^13^

The cure rate in this report showed consistent increase in the proportion of patients achieving an MI of 0 following the addition of ofloxacin, demonstrating the treatment’s effectiveness in reducing viable bacterial load over time. This result is similar to other study evaluating OMDT in non-endemic area that showed a high cure rate and tolerability. ^5^ Other studies evaluating the effectivity of ofloxacin combined with rifampicin and minocycline showed 2-year cure rate which achieved 93.1% to 99%. ^14, 15^ Moreover, a clinical trial that followed up for an average 10.8 years after ofloxacin-containing regimen, reported relapse in one out of 58 patients. ^16^ These findings supported that adding ofloxacin might be a promising alternative to standard WHO MDT, due to the high cure rate reported.

Ofloxacin offers a notable advantage in leprosy treatment due to its rapid onset of action, surpassing the effects of several conventional drugs. Studies indicate its efficacy in quickly decreasing the bacterial index (BI), a crucial indicator on monitoring severe or drug resistant leprosy cases. ^17^ This study’s findings confirm the established efficacy of ofloxacin, demonstrating a significant reduction in the MI, which reflects the proportion of viable bacilli, after the addition to the standard MDT regimen. Specifically, the median MI was observed to reach zero within six months of treatment initiation. The statistical analysis confirmed significant differences in MI across the assessed time points (p < 0.001). Subsequent post-hoc analysis revealed significant differences between baseline and six months (p = 0.003), nine months (p = 0.014), and 12 months (p = 0.008). Previous studies showed ofloxacin daily on lepromatous leprosy patients produced remarkable clinical improvement, rapid and significant decline of the MI, and killing of > 99 and > 99.99% of the viable M. leprae present before treatment by 14 and 28 days, respectively. ^18^ This is also consistent to the previous observation that administration of ofloxacin resulted in the complete inhibition of bacterial multiplication and no resumption of growth was observed even 18 months after treatment. ^19^ Another study shows that, compared to MDT-WHO regimen, the decrease of MI score of patients treated with ofloxacin combined with rifampicin and minocycline is more significant, 79.97% of WHO MDT and 94.83% of ROM regimen. ^20^

In addition to MI, our study also observed consistent and significant reductions in the bacterial index (BI) following the addition of ofloxacin. Significant differences were noted across visits (p = 0.007), with pairwise comparisons showing significant reductions in BI between baseline and three months (p = 0.039), six months (p = 0.003), nine months (p = 0.006), and 12 months (p = 0.004). These findings emphasize the importance of adding ofloxacin to MDT regimens as early as possible, particularly in cases with a high bacterial load or when certain clinical criteria are met. Rapid reductions in both MI and BI not only reflect effective bacterial clearance but also reduce the risk of relapse and disease transmission. ^7^

Our data suggests that early initiation of ofloxacin, particularly with inclusion criteria as mentioned above, may lead to faster bacterial clearance and improved patient outcomes. Notably, in our study, patients received WHO-MDT for a duration of 9 months (median; range 1-31 months) before starting combined therapy with ofloxacin. However, waiting for 9 months or longer may not be necessary. If the BI and MI are very high, the addition of ofloxacin should be considered as early as possible to achieve a faster decline in BI and MI, thereby shortening the treatment duration, improving adherence, and reducing the risk of treatment discontinuation.

Ofloxacin not only enhances bactericidal efficacy but also prevents drug resistance when combined with other antibiotics such as rifampicin, clofazimine, dapsone. This combination also ensures a broad coverage against the bacterium, improving overall treatment outcomes while reducing the risk of resistance, a major concern in long-term leprosy management. ^21^ Moreover, studies have demonstrated that ofloxacin combined with rifampicin and minocycline produces fewer side effects compared to the MDT-WHO regimen, which may improve patient adherence and treatment success rate. ^20^

The broader application of ofloxacin is particularly crucial in MB leprosy, where the bacterial load and MI are higher. ^22^ Studies have demonstrated that combining ofloxacin with minocycline have been shown to be more bactericidal than dapsone and clofazimine in both mice in both mice and clinical trial. ^23^ This enhanced efficacy underlines the necessity of incorporating ofloxacin into treatment regimens especially in special cases, for instance, the dapsone hypersensitivity syndrome (DHS). ^24^ DHS is a complication where patients are unable to tolerate dapsone as part of standard MDT. In such cases, the addition of ofloxacin provides a viable alternative to ensure effective bacterial clearance. This clearance is crucial as high BI is one of the risk factors for the occurrence of grade 2 disability. ^25^

Finally, in cases of drug-resistant leprosy, ofloxacin has been used as an alternative when traditional drugs like dapsone or clofazimine are ineffective. ^5^ This makes it a critical option in the arsenal against resistant strains of *M. leprae*, providing hope for patients who may not respond to standard therapies. As leprosy cases become increasingly concentrated in specific regions, the role of newer antibiotics like ofloxacin will likely expand to support global efforts in reducing disease prevalence and preventing disabilities associated with untreated leprosy. ^20^

This is the first study from Indonesia to evaluate the effectiveness of adding ofloxacin to the standard WHO MDT regimen for leprosy. It is important to note that this approach is applicable only for certain cases, particularly lepromatous form of leprosy patients who meet the following criteria: (1) a high initial viable infection load, as evidenced by a high morphological index (MI) from acid-fast staining of slit-skin smears; (2) persistently positive MI, typically after ≥6 months of standard MDT; (3) re-positivity of MI; and (4) relapse, diagnosed based on criteria by Linder et al. ^8^

In Indonesia, ofloxacin is currently the only second-line treatment for leprosy covered by the national health insurance system. However, its use is restricted to the national referral hospital and is covered only for one week-long of administration. For the next three weeks of consumption, patients are required to either purchase the medication themselves or visit the hospital weekly to receive it. This limitation emphasizes the need for policy changes to improve access to this promising therapy, particularly for patients in rural areas.

We acknowledge that this study was conducted retrospectively and has limitations, including potential for biases to inherent to this design. Additionally, the safety and drug tolerance profile regarding ofloxacin may not have been adequately captured due to limited documentation in these records. Despite these limitations, we believe that our findings could be a preliminary data for the future randomized control trial study with larger sample size to evaluate the efficacy of ofloxacin in lepromatous form of leprosy thoroughly.

## Conclusion

Addition of ofloxacin to standard MDT WHO in lepromatous form of leprosy patients significantly reduces both in the MI and BI after six months and three months, respectively. Addition of ofloxacin also demonstrated the treatment’s effectiveness in reducing viable bacterial load over time.

## Ethics and consent

The study protocol was reviewed and approved by the Ethics Committee of the Faculty of Medicine, Universitas Indonesia – Dr. Cipto Mangunkusumo Hospital (Komite Etik Penelitian Kesehatan Fakultas Kedokteran Universitas Indonesia – RSUPN Dr. Cipto Mangunkusumo).

The approval date was December 8, 2024, valid for one year from the date of approval. The protocol number assigned to this study is KET-1769/UN2.F1/ETIK/PPM.00.02/2024. As this research involved retrospective secondary data extracted from medical records, no direct patient contact was required. Consequently, the need for individual informed consent specific to this study was waived by the Ethics Committee. However, all patients at Dr. Cipto Mangunkusumo Hospital provide general consent for treatment and the use of their data for research purposes upon admission, with strict adherence to anonymity, and in accordance with all relevant regulations and ethical standards.

## Contributors

MHP: Conceptualisation, data curation, methodology, statistical analysis, visualisation, writing (original draft and revisions)

MSY: Data curation, methodology, statistical analysis, visualisation, writing (original draft and revisions)

SLM: Conceptualisation, data curation, methodology, writing (original draft and revisions)

EJN: Conceptualisation, data curation, methodology, writing (original draft and revisions)

MM: Conceptualisation, data curation, methodology, writing (original draft and revisions)

## Data Availability

Figshare: Dataset OMDT.xlsx. https://doi.org/10.6084/m9.figshare.28331675.v1. ^26^ The project contains the following underlying data:
• Dataset OMDT.xlsx Data are available under the terms of the Creative Commons Attribution 4.0 International license (CC-BY 4.0).

## Software availability

To facilitate accessibility for future studies, we note the following open-source alternatives, such as JASP (JASP Team, Amsterdam, Netherlands) for statistical analyses and ggplot2 3.5.1 (Hadley Wickham, Auckland, New Zealand) or SciDAVis (SciDAVis Development Team) for data visualization.
