# Supplementary material for: Optimizing treatment of lepromatous form of leprosy using ofloxacin on top of standard multi-drug therapy in National Referral Hospital, Jakarta, Indonesia
Source: F1000Res. 2025 Oct 3;14:252. Originally published 2025 Mar 3. [Version 4] doi: 10.12688/f1000research.161758.4 (PMC12552836; doi:10.12688/f1000research.161758.4)
Supplement: Peer review history

# Reviewer report: Reviewer response for version 4

**DOI:** 10.5256/f1000research.188786.r421048

**Published:** 2025-11-20

**Authors:** Hongsheng Wang

**Affiliations:** 1 Nanjing Medical University, Nanjing, China

It is recommended to classify the treatment outcomes of patients according to the following baseline conditions:

 “(a) a high initial viable infection load, evidenced by a high MI from acid-fast staining of slit-skin smears; (b) persistently positive MI, typically after ≥6 months of standard MDT; (c) re-positivity of MI; and (d) relapse.”

 Regarding Figure 2, the sample size at each time point decreased significantly (n = 21 → 15 → 10 → 7).

 The authors only compared MI and BI at each follow-up point with the baseline, which may represent inconsistent comparison sets. Were there any cases with a 6-month follow-up but without a 3-month follow-up?

 It is suggested to also perform comparisons between different time points (e.g., 3 vs 6 months) to avoid missing information related to declining trends and relapse.

 After reaching MI = 0, was continuous follow-up performed? Were there any cases of “(c) re-positivity of MI” or “(d) relapse”?

 Using only */ns to indicate significance is insufficient to reflect biological or clinical relevance; median values and 95% confidence intervals should be reported to better interpret the actual magnitude of change.

 Were there any cases that did not take ofloxacin, used other medications, or received extended MDT regimens? It is recommended to include these cases for comparison.

 Were there any leprosy reactions during treatment? Were any adverse effects of ofloxacin observed? It is suggested to add relevant descriptions.

Is the work clearly and accurately presented and does it cite the current literature?

Yes

If applicable, is the statistical analysis and its interpretation appropriate?

Partly

Are all the source data underlying the results available to ensure full reproducibility?

No source data required

Is the study design appropriate and is the work technically sound?

Partly

Are the conclusions drawn adequately supported by the results?

Partly

Are sufficient details of methods and analysis provided to allow replication by others?

Yes

Reviewer Expertise:

leprosy control

I confirm that I have read this submission and believe that I have an appropriate level of expertise to confirm that it is of an acceptable scientific standard, however I have significant reservations, as outlined above.

# Reviewer report: Reviewer response for version 4

**DOI:** 10.5256/f1000research.188786.r421040

**Published:** 2025-10-25

**Authors:** Bruno de Carvalho Dornelas

**Affiliations:** 1 Federal University of Uberlândia,, Uberlândia,, Brazil

This study, a single-center retrospective cohort analysis, investigates the efficacy of adding ofloxacin (400 mg once daily) to the standard WHO Multi-Drug Therapy (MDT) regimen for patients diagnosed with lepromatous (LL) or borderline lepromatous (BL) leprosy (N=21). The primary objective was to assess the impact of this adjunctive therapy on bacteriological outcomes, specifically measuring the Morphological Index (MI) and the Bacteriological Index (BI). The authors found a significant reduction in both MI and BI after the addition of ofloxacin, noting that the median MI dropped to zero after six months of combined treatment. This outcome is interpreted as supporting the potent bactericidal activity of ofloxacin, leading to faster bacterial clearance in high-bacterial-load cases, potentially enhancing adherence and reducing relapse risk.

*Standard multi-drug therapy (MDT) with duration of 6-12 months is generally effective for treating leprosy*. WHO/MDT dates from 80s. We need new drugs as ofloxacin. Relapse and treatment failure rates are not low. Could you please cite: [Nascimento ACMD, et al (2022) - Ref 1]

*Despite global efforts to eliminate Leprosy*. Please, keep it in lowercase (leprosy).

*where the bacterial load is high and conventional MDT may take longer time than standard duration of multibacillary (MB) therapy to achieve bacteriological cure*. How do you define bacteriological cure?

 Please, exclusion criteria do not make sense. Having the available data in an inclusion criterion, isn’t it? Exclusion criteria are not the opposite of those. Maybe, patients with other infectious disease were exclude…. Please, note this.

 At each visit, dermatology resident? No supervision from a senior dermatologist?

 Nearly half of the initial patients (N=43) were excluded due to missing data or failure to return for follow-up. This high attrition rate in a retrospective single-center study limits the generalizability of the findings to broader populations and introduces potential selection bias. This limitation is noted by the authors and should be continuously emphasized. For future research, as the authors mention, strategies must be implemented to ensure prospective data collection and patient retention for long-term chronic disease follow-up.

 The work addresses a clinically relevant challenge: the optimization of treatment for highly bacilliferous forms of leprosy (LL/BL) where standard MDT alone may lead to prolonged viable infection and potential issues with adherence or relapse. The use of BI and MI as primary bacteriological outcomes is appropriate for assessing treatment efficacy.

 However, as highlighted by multiple reviewers, the study design presents significant methodological limitations that restrict the strength and generalizability of the conclusions. The study primarily measures change within the same cohort *after* the introduction of ofloxacin, making it difficult to definitively attribute the acceleration of clearance entirely to the added drug, rather than the ongoing effects of standard MDT.

 The most substantial weakness is the absence of a concurrent control group receiving standard MDT without ofloxacin. This retrospective design limits the ability to compare outcomes directly, preventing the authors from asserting that the observed acceleration in bacterial clearance (MI and BI reduction) is significantly faster than what would have occurred naturally with standard MDT in this specific population.

 While the authors explained that ethical considerations at a national referral center precluded withholding potentially beneficial treatment from high-risk patients solely for a comparison group, this needs to be emphasized more strongly as a limitation when interpreting results. The language used in the Abstract—suggesting the approach helps "prevent prolonged treatment duration"—must be tempered, as the Discussion noted that the overall duration remained similar, but the efficacy was enhanced. The authors should clarify the focus: is the main goal shortening the duration, or achieving bacteriological cure faster within the standard timeframe?

*The primary outcomes of this study are (1) change in Bacteriological Index (BI) as this measures the bacterial load in skin smears, and (2) change in Morphological Index (MI)*. Please, once abbreviated, keep using abbreviations throughout the text.

*The majority of the subjects were male (66.7%) and unemployed (42.9%). *Please, rewrite this. 42,9% is not the majority, is it?

 The monitoring of adverse effects was primarily passive, relying on documentation in medical records if patients reported complaints, rather than a systematic evaluation. This is inadequate, especially given that ofloxacin is known to cause serious side effects like tendinopathy and peripheral neuropathy. The absence of documentation does not guarantee the absence of adverse effects.

 Future, more thorough studies should incorporate a systematic and active monitoring protocol for known adverse effects associated with ofloxacin, particularly musculoskeletal and neurological complications.

*Studies of the rifampicin-ofloxacin-minocycline (ROM) combination reported 2-year cure rate ranging from 93.1% to 99%. *What is cure in leprosy? What are the criteria for it? I am not aware of any.

 Ofloxacin was initiated under circumstances including a "high initial viable infection load, evidenced by a high MI".  The authors should explicitly define the internal threshold or clinical criteria used that qualified an MI result as "high" enough to warrant immediate addition of second-line therapy, thereby providing crucial context for replication.

 The article presents useful preliminary data supporting the use of ofloxacin as an adjunctive treatment for high-bacterial-load leprosy, demonstrating faster bacteriological clearance. The importance of optimizing treatment for these challenging cases is clear. However, significant methodological limitations inherent to the retrospective design and the passive nature of adverse effects monitoring weaken the conclusiveness and generalizability of the results.

 Please:

 1. Ensure the interpretation of the findings consistently reflects the limitations inherent to the retrospective, single-center, non-controlled design.

 2. Explicitly define the criteria used by the center to determine a "high MI" that justified the addition of ofloxacin to the standard MDT.

 3. Ensure the Introduction/Abstract clearly states that the benefit of Ofloxacin is accelerating clearance, rather than definitively preventing prolonged treatment duration, unless the data unequivocally supports a shorter course.

Is the work clearly and accurately presented and does it cite the current literature?

Yes

If applicable, is the statistical analysis and its interpretation appropriate?

Yes

Are all the source data underlying the results available to ensure full reproducibility?

Partly

Is the study design appropriate and is the work technically sound?

Partly

Are the conclusions drawn adequately supported by the results?

Partly

Are sufficient details of methods and analysis provided to allow replication by others?

Yes

Reviewer Expertise:

Pathology and epidemiology and leprosy

I confirm that I have read this submission and believe that I have an appropriate level of expertise to confirm that it is of an acceptable scientific standard, however I have significant reservations, as outlined above.

## References

[1] : Leprosy Relapse: A Retrospective Study on Epidemiologic, Clinical, and Therapeutic Aspects at a Brazilian Referral Center. *International Journal of Infectious Diseases* .2022;118: 10.1016/j.ijid.2022.01.009 44-51 10.1016/j.ijid.2022.01.009 35017109

[2] : Role of histopathological, serological and molecular findings for the early diagnosis of treatment failure in leprosy. *BMC Infectious Diseases* .2024;24(1) : 10.1186/s12879-024-09937-2 10.1186/s12879-024-09937-2 39354399 PMC11443919

[3] : Impact of histopathological and serological assessments on early diagnosis of leprosy relapse. *APMIS* .2025;133(1) : 10.1111/apm.13497 10.1111/apm.13497 39530153

# Reviewer report: Reviewer response for version 4

**DOI:** 10.5256/f1000research.188786.r421045

**Published:** 2025-10-25

**Authors:** Isadora Costa Celestino

**Affiliations:** 1 Federal University of Uberlândia, Uberlândia, Brazil

I believe the study was conducted appropriately, considering its design, and that important points regarding the efficacy of ofloxacin combined with MDT were discussed, particularly the report of a decrease in BI and MI over time. However, I would like to make a few observations:

 1. In the paragraph "A retrospective study further demonstrated that patients receiving MDT combined with ofloxacin showed good adherence, but no adherence failures were identified among subjects receiving ofloxacin. Additionally, the combination therapy was associated with a lower relapse rate and improved long-term outcomes, supporting its use as a promising option for treating severe leprosy cases."5, the study "Faust L, et al., 2021 [Ref- 1]" did not conclude that the use of ofloxacin increases adherence. Rather, it indicated that OMDT helped to reduce some of the existing barriers related to medication, compared to the numerous ones usually encountered.

 2. What was the MI cutoff (in percentage) used to define "a high initial viable infection load, evidenced by a high MI from acid-fast staining of slit-skin smears"? I suggest making this explicit.

 3. Were there no adverse reactions described in the records? If not, I suggest mentioning this explicitly in the results. Even if this absence is part of the study design as a bias, the methodology section indicates that adverse reaction were monitored. Therefore, it would be helpful to clearly state that no adverse reactions were observed.

 I believe that once the authors evaluate these points, the article will be even more complete.

Is the work clearly and accurately presented and does it cite the current literature?

Yes

If applicable, is the statistical analysis and its interpretation appropriate?

Yes

Are all the source data underlying the results available to ensure full reproducibility?

Yes

Is the study design appropriate and is the work technically sound?

Yes

Are the conclusions drawn adequately supported by the results?

Yes

Are sufficient details of methods and analysis provided to allow replication by others?

Yes

Reviewer Expertise:

Pharmacovigilance, Leprosy, Clinical Pharmacy, Pharmacology.

I confirm that I have read this submission and believe that I have an appropriate level of expertise to confirm that it is of an acceptable scientific standard.

## References

[1] : Ofloxacin-Containing Multidrug Therapy in Ambulatory Leprosy Patients: A Case Series. *Journal of Cutaneous Medicine and Surgery* .2021;25(1) : 10.1177/1203475420952437 45-52 10.1177/1203475420952437 32869655

# Reviewer report: Reviewer response for version 3

**DOI:** 10.5256/f1000research.186135.r407739

**Published:** 2025-09-03

**Authors:** Fifa Argentina

**Affiliations:** 1 Universitas Sriwijaya, Palembang, South Sumatra, Indonesia

The study highlights ofloxacin’s potential to accelerate bacteriological clearance in lepromatous leprosy, but methodological issues limit its strength. Lack of a control group, retrospective single-center design, and substantial missing follow-up reduce generalizability. Discussion remains repetitive, adverse event monitoring was inadequate, and inconsistencies in patient selection should be clarified. Thank you.

Is the work clearly and accurately presented and does it cite the current literature?

Yes

If applicable, is the statistical analysis and its interpretation appropriate?

Yes

Are all the source data underlying the results available to ensure full reproducibility?

Yes

Is the study design appropriate and is the work technically sound?

Yes

Are the conclusions drawn adequately supported by the results?

Yes

Are sufficient details of methods and analysis provided to allow replication by others?

Yes

Reviewer Expertise:

The use of standard leprosy drugs in some cases is often resistant, characterized by unchanged bacterial index and morphology index values or only a slight decrease. This results in longer MDT drug use and reduced patient compliance with medication. With preliminary research on the use of ofloxacin, it can be used as a guideline for further research in the future.

I confirm that I have read this submission and believe that I have an appropriate level of expertise to confirm that it is of an acceptable scientific standard.

# Response

**Published:** 2025-09-25

**Authors:** Mufqi Handaru Priyanto

**Affiliations:** Dermatology and Venereology, Universitas Indonesia, Depok, West Java, Indonesia

We thank the reviewer for the constructive comments. Concerning the absence of a control group and the retrospective single-center design, we acknowledge that this limits the generalizability of the findings; this has already been addressed in the Limitations section, and we have strengthened our interpretation by referencing comparable published studies. Regarding adverse event monitoring, we would like to clarify that side effects were monitored through patient medical records during follow-up visits. Any adverse events related to treatment would have been documented in the patient’s status notes, while the absence of such documentation indicated no adverse events. To make this explicit, we have added a statement in the Methods section specifying that adverse event monitoring was integrated into clinical follow-up and recorded accordingly. In addition, the Discussion section has been revised to reduce repetition and improve clarity while maintaining alignment with the cited literature. We believe these revisions address the reviewer’s concerns and strengthen the manuscript

# Reviewer report: Reviewer response for version 2

**DOI:** 10.5256/f1000research.180812.r384857

**Published:** 2025-06-13

**Authors:** Brandon L Adler

**Affiliations:** 1 Department of Dermatology, Keck School of Medicine, University of Southern California, Los Angeles, California, USA

Thank you for this study evaluating the impact of addition of ofloxacin to MDT for lepromatous leprosy, conducted in a single center.

 “At our center, slit-skin smear examinations are routinely performed from six sites (both earlobes and four lesions) every three months in patients with positive initial MI or in cases requiring close monitoring.” Accordingly, there seems to be no reason that a cohort of patients receiving MDT without ofloxacin from your center could be compared to those receiving ofloxacin with regard to the BI and MI.  That is what is missing for this study to be truly clinically meaningful.

 In the abstract you state, “As prolonged duration may affect patient adherence negatively, adding ofloxacin to MDT is a promising approach to prevent prolonged treatment.” In the discussion: “Though the treatment duration remains similar to standard MDT, ofloxacin-added regimens may offer enhanced efficacy, particularly in lepromatous cases.” These statements seem contradictory, and maybe the one in the abstract should be toned down?

 Statistical methods (non-parametric Friedman test) can be deleted from the study abstract.

 Methods: “The most common adverse effect of this drug is tendinopathy, and there may be hypersensitivity reactions, central nervous system adverse drug reactions, peripheral neuropathy, though uncommon.” This is not a study method and does not belong in this section of the paper. It can go in the introduction (assuming you report the numbers of patients developing side effects to ofloxacin) or in the discussion (since it seems side effects were not evaluated- which is another limitation).

 In figure 1, I don’t understand how 3 patients were excluded due to not having BL/LL leprosy, if the initial N=43 includes only lepromatous patients. Also in figure 1, we can see that nearly half of patients were excluded due to missing data or not returning for follow up, a major limitation.

 Figure 2 showing the map of patients’ domiciles is extraneous to the study aims and can be deleted.

Is the work clearly and accurately presented and does it cite the current literature?

Partly

If applicable, is the statistical analysis and its interpretation appropriate?

Yes

Are all the source data underlying the results available to ensure full reproducibility?

No

Is the study design appropriate and is the work technically sound?

No

Are the conclusions drawn adequately supported by the results?

Partly

Are sufficient details of methods and analysis provided to allow replication by others?

Yes

Reviewer Expertise:

leprosy

I confirm that I have read this submission and believe that I have an appropriate level of expertise to state that I do not consider it to be of an acceptable scientific standard, for reasons outlined above.

# Response

**Published:** 2025-08-04

**Authors:** Mufqi Handaru Priyanto

**Affiliations:** Dermatology and Venereology, Universitas Indonesia, Depok, West Java, Indonesia

Thank you for this study evaluating the impact of addition of ofloxacin to MDT for lepromatous leprosy, conducted in a single center.

  

1. “At our center, slit-skin smear examinations are routinely performed from six sites (both earlobes and four lesions) every three months in patients with positive initial MI or in cases requiring close monitoring.” Accordingly, there seems to be no reason that a cohort of patients receiving MDT without ofloxacin from your center could be compared to those receiving ofloxacin with regard to the BI and MI.  That is what is missing for this study to be truly clinically meaningful.

Thank you for this observation. We acknowledge that the inclusion of a control group receiving standard MDT without ofloxacin would indeed strengthen the study's design and enhance its clinical meaningfulness, allowing for a more direct comparison of bacteriological indices (BI) and morphological indices (MI) changes. However, it is crucial to clarify the ethical and practical considerations at our center that led to the current study design. Our institution, as a national referral hospital, primarily manages complex and challenging cases of leprosy, including those with high initial MI, persistent positivity, relapse, or re-positivity, where the standard MDT alone might be insufficient to achieve optimal clinical and bacteriological clearance within the conventional timeframe. The decision to add ofloxacin in these specific cases is a clinical judgment made in accordance with the severity of the disease and the need for more aggressive bacteriological clearance to prevent further complications and transmission. Therefore, withholding an additional, potentially beneficial, antimicrobial agent from these high-risk patients solely for research comparison was considered as ethically unfeasible, given the established clinical practice and patient well-being priorities.

 While a concurrent control group was not feasible, we aimed to demonstrate the efficacy of ofloxacin-added regimens within this cohort by observing significant reductions in MI and BI over time, and by comparing these outcomes against the expected prolonged treatment durations typically observed in such cases under standard MDT alone. We agree that future multi-center studies, perhaps with different patient populations or in settings where such a comparative arm is ethically permissible, would be invaluable to further validate our findings.

  

1. In the abstract you state, “As prolonged duration may affect patient adherence negatively, adding ofloxacin to MDT is a promising approach to prevent prolonged treatment.” In the discussion: “Though the treatment duration remains similar to standard MDT, ofloxacin-added regimens may offer enhanced efficacy, particularly in lepromatous cases.” These statements seem contradictory, and maybe the one in the abstract should be toned down?

We appreciate you highlighting this discrepancy. You are correct that the statements regarding treatment duration could appear contradictory. We have revised the abstract to better reflect the findings and align with the discussion.

 In the abstract, our initial *statement 'As prolonged duration may affect patient adherence negatively, adding ofloxacin to MDT is a promising approach to prevent prolonged treatment'* was an initial hypothesis and a general clinical rationale. However, as revealed in our results and discussed in detail, the addition of ofloxacin did not, in this specific cohort, *shorten* the overall treatment duration compared to the *minimum* standard MDT for lepromatous leprosy. Instead, its benefit lies in *accelerating bacteriological clearance* within that standard duration, which is crucial for preventing relapse and reducing transmission, especially in difficult-to-treat cases.

 Therefore, we have revised the abstract statement on the newest version.

 2. Statistical methods (non-parametric Friedman test) can be deleted from the study abstract. We concur with your suggestion. The specific mention of 'non-parametric Friedman test' in the abstract is indeed overly detailed for this section. We have removed it from the abstract to maintain conciseness and focus on the primary findings, while ensuring the full details of our statistical analysis remain in the Methods section.

 3. Methods: “The most common adverse effect of this drug is tendinopathy, and there may be hypersensitivity reactions, central nervous system adverse drug reactions, peripheral neuropathy, though uncommon.” This is not a study method and does not belong in this section of the paper. It can go in the introduction (assuming you report the numbers of patients developing side effects to ofloxacin) or in the discussion (since it seems side effects were not evaluated- which is another limitation). We appreciate this astute observation regarding the placement of the adverse effect description. You are absolutely correct that this information does not belong in the Methods section, which should be reserved for how the study was conducted.

 We have moved the description of ofloxacin's common adverse effects, including tendinopathy, hypersensitivity reactions, CNS adverse drug reactions, and peripheral neuropathy, to the **Introduction section**. This provides essential background information for readers regarding the drug's safety profile before they delve into our findings.

 Regarding the evaluation of side effects in our study, we acknowledge this as a limitation. While we did not conduct a systematic, pre-specified evaluation of all potential adverse effects, any reported side effects during treatment were documented in patient medical records. However, due to the retrospective nature of some data collection and the primary focus on bacteriological outcomes, a comprehensive analysis of adverse events was beyond the scope of this particular study. We have explicitly stated this as a limitation in the Discussion section, consistent with your feedback.

 4. In figure 1, I don’t understand how 3 patients were excluded due to not having BL/LL leprosy, if the initial N=43 includes only lepromatous patients. Also in figure 1, we can see that nearly half of patients were excluded due to missing data or not returning for follow up, a major limitation. 

 Thank you for pointing out the inconsistencies in Figure 1. We apologize for the confusion.

 Regarding the initial cohort of 43 patients and the subsequent exclusions, it is important to clarify our patient selection process. The initial N=43 represents all leprosy patients who received ofloxacin in addition to standard MDT at our center during the study period, as identified from our pharmacy records. From this initial pool, patients were then filtered based on our specific inclusion criteria for this study, which required a diagnosis of lepromatous leprosy (BL/LL). During a re-review of their detailed clinical and histopathological records for this specific study, 3 patients, despite an initial classification, were found to have clinical features and biopsy results that did not unequivocally meet the criteria for the lepromatous (BL/LL) spectrum as defined for our study's inclusion. We have revised the flowchart to clarify this specific reason for exclusion.

 We fully agree with your observation that the exclusion of nearly half of the patients due to missing data or not returning for follow-up represents a significant limitation. We have acknowledged and emphasized this point more strongly in our Discussion section. This high reduction rate is indeed a challenge inherent to real-world clinical data collection in a referral setting, particularly for chronic diseases requiring long-term follow-up. While regrettable, we believe the remaining robust dataset still provides valuable insights into the efficacy of ofloxacin in the responsive patients within this challenging group. We are actively working on strategies to improve patient retention and data completeness in future studies.

 5. Figure 2 showing the map of patients’ domiciles is extraneous to the study aims and can be deleted.

 We concur with your assessment that Figure 2 is extraneous to the core aims of this study, which primarily focus on the clinical and bacteriological outcomes of the treatment regimen. We have removed Figure 2 from the manuscript.

# Reviewer report: Reviewer response for version 1

**DOI:** 10.5256/f1000research.177837.r369473

**Published:** 2025-04-02

**Authors:** Maria T Ochoa

**Affiliations:** 1 Department of Dermatology, Keck School of Medicine, University of Southern California, Los Angeles, Southern California, USA

The work done by Priyanto al is interesting and in my opinion the article adds new insights into the treatment of leprosy in Indonesia

 The article needs improvements in the methods, results and discussion.

 - The authors should include a cohort of patients treated with multidrug therapy without ofloxacin. That will improve the quality of the paper

 -Authors should explain the meaning of  “high initial viable infection load, evidenced by a high MI” what is considered a high MI

 -Authors should mention the main dose used for ofloxacin and also the main side effects of this medication.

 - The discussion is too long, very repetitive and should be reduced.

 The findings are interesting, however the authors cannot draw conclusions. The authors need to acknowledge this limitation.

Is the work clearly and accurately presented and does it cite the current literature?

Partly

If applicable, is the statistical analysis and its interpretation appropriate?

Yes

Are all the source data underlying the results available to ensure full reproducibility?

Yes

Is the study design appropriate and is the work technically sound?

No

Are the conclusions drawn adequately supported by the results?

Partly

Are sufficient details of methods and analysis provided to allow replication by others?

No

Reviewer Expertise:

leprosy

I confirm that I have read this submission and believe that I have an appropriate level of expertise to confirm that it is of an acceptable scientific standard, however I have significant reservations, as outlined above.

# Response

**Published:** 2025-04-17

**Authors:** Mufqi Handaru Priyanto

**Affiliations:** Dermatology and Venereology, Universitas Indonesia, Depok, West Java, Indonesia

We sincerely appreciate the reviewer’s constructive feedback on our manuscript. Below, we address each of the points raised:

 1. Regarding the suggestion to include a cohort treated without ofloxacin:

 We acknowledge the importance of this comparison. However, as this is a preliminary study, our aim was to analyze treatment outcomes before and after the addition of ofloxacin within the same patient group. Should the current findings prove promising, a subsequent study involving a direct comparison between ofloxacin and non-ofloxacin groups will be conducted to strengthen this finding 

 2. Regarding the clarification of “high initial viable infection load, evidenced by a high MI”:

 A high MI (Morphological Index) was not the sole criterion for patient recruitment in this study. While there is currently no universally accepted threshold in the international literature defining what constitutes a "high" MI, we based our observations on patient data from our center. In addition to elevated MI values, other factors such as persistent positivity, relapse, and re-positivity were also considered in selecting subjects for inclusion.

 3. Regarding the main dose and side effects of ofloxacin:

 We have now included a description of the dose of ofloxacin along with its commonly reported side effects

 4. Regarding the discussion section:

 We have revised the discussion section to make it more concise

 Thank you so much for your time
